# Comparative Effectiveness of Sodium-Glucose Cotransporter-2 (SGLT2) Inhibitors Versus Glucagon-Like Peptide-1 (GLP-1) Agonists on Cardiovascular and Renal Outcomes in Type 2 Diabetes: A Systematic Review and Network Meta-Analysis

**DOI:** 10.7759/cureus.100927

**Published:** 2026-01-06

**Authors:** Aymen A Alqurain, Manal Salem, Bashayer A Algarzai, Eman M Eljadi, Hazem S Elsayed, Ahmed Almuhanna, Deema S Albuhayji, Mohammad Salami, Joud Alzahrani, Juri Alsayyali, Abdulrahman Alqarni, Nessreen Alqahtani, Fay T Alotaibi, Gharam G Alyami

**Affiliations:** 1 Clinical Practice, Faculty of Pharmacy, Northern Border University, Rafha, SAU; 2 Medical Education and Simulation, Al Baha University, Al-Baha, SAU; 3 Pharmacy, Qassim University, Al-Qassim, SAU; 4 Clinical Pharmacy, Misurata Health Services Center, Misurata, LBY; 5 Clinical Pharmacy, Cairo University Hospitals, Cairo, EGY; 6 Clinical Pharmacy, King Faisal University, Al-Ahsa, SAU; 7 Pharmacy, King Abdulaziz Hospital, Ministry of National Guard - Health Affairs, Al-Ahsa, SAU; 8 Medical School, King Saud Bin Abdulaziz University for Health Sciences, Riyadh, SAU; 9 Medicine and Surgery, King Abdulaziz University, Jeddah, SAU; 10 General Practice, Ministry of Defense (MOD), Tabuk, SAU; 11 Nursing, King Abdulaziz University Hospital, Jeddah, SAU; 12 Pharmacy, Shaqra University, Shaqra, SAU; 13 Pharmacy, Princess Nourah Bint Abdulrahman University, Riyadh, SAU

**Keywords:** cardiovascular outcomes, chronic kidney disease, comparative effectiveness, glp-1 receptor agonists, heart failure, network meta-analysis, sglt2 inhibitors, type 2 diabetes

## Abstract

Sodium-glucose cotransporter-2 (SGLT2) inhibitors and glucagon-like peptide-1 (GLP-1) receptor agonists reduce cardiovascular and renal risks in type 2 diabetes mellitus (T2DM), but their relative efficacy remains uncertain due to the absence of direct comparative trials. This systematic review and network meta-analysis aimed to evaluate the efficacy and safety of interventions concerning major adverse cardiovascular events (MACE), heart failure, and renal outcomes. A systematic review and network meta-analysis of large-scale, placebo-controlled cardiovascular outcome trials was conducted. PubMed, Embase, and CENTRAL were searched for trials published up to December 2025. The primary outcome was MACE (cardiovascular death, nonfatal myocardial infarction, or nonfatal stroke). The secondary outcomes included hospitalization for heart failure (HHF), composite renal outcomes, and all-cause mortality. The evaluated safety outcomes included severe hypoglycemia, diabetic ketoacidosis, amputation, fracture, and genital infections. Data were pooled using a frequentist random-effects model. In total, 14 trials involving 117,633 participants were included. Both drug classes reduced the risk of MACE compared with placebo (SGLT2i: hazard ratio (HR) = 0.89, 95% confidence interval (CI) = 0.84-0.94; GLP-1RA: HR = 0.86, 95% CI = 0.80-0.93), with no statistically significant difference observed between the two (HR = 1.03, 95% CI = 0.94-1.13). SGLT2 inhibitors had a greater efficacy than GLP-1 receptor agonists in reducing HHF (HR = 0.75, 95% CI = 0.66-0.85) and composite renal outcomes (HR = 0.76, 95% CI = 0.66-0.87). Similarly, both classes lowered all-cause mortality. SGLT2 inhibitors exhibited an elevated risk of genital infections (relative risk (RR) = 3.49, 95% CI = 2.63-4.55) and diabetic ketoacidosis (RR = 2.36, 95% CI = 1.33-4.17) compared to GLP-1 receptor agonists. SGLT2 inhibitors and GLP-1 receptor agonists are equally effective in preventing MACE. However, SGLT2 inhibitors offer enhanced protection against heart failure and renal disease progression, whereas GLP-1 receptor agonists exhibit a more favorable safety profile for genital infections and ketoacidosis. These findings support a phenotype-specific treatment approach for patients with T2DM.

## Introduction and background

Type 2 diabetes mellitus (T2DM) is a rapidly expanding health emergency. The 11th Edition of the International Diabetes Federation Diabetes Atlas estimates that 589 million adults worldwide had diabetes, with a projection indicating an increase to 853 million by 2050 [1]. T2DM is associated with an increased risk of macrovascular and microvascular complications, which are the leading causes of morbidity and mortality in this population. Specifically, the coexistence of cardiovascular disease (CVD) and chronic kidney disease (CKD) exerts a synergistic deleterious effect on patient prognosis, requiring therapeutic strategies that go beyond glucose-lowering to offer comprehensive cardiorenal protection [2,3].

In the last decade, T2DM therapy has shifted with the advent of sodium-glucose cotransporter-2 (SGLT2) inhibitors and glucagon-like peptide-1 (GLP-1) receptor agonists as these agents have shown significant cardioprotective and renoprotective benefits in large-scale cardiovascular outcome trials (CVOTs) [4]. These classes appear to diverge as SGLT2 inhibitors exert hemodynamic effects, including natriuresis and reduction of intraglomerular pressure, whereas GLP-1 receptor agonists are posited to mitigate atherosclerotic risk through metabolic and anti-inflammatory mechanisms, including weight reduction, improved insulin sensitivity, and the attenuation of vascular inflammation [3,5].

International guidelines recommend these medications as first-line therapies for patients with T2DM and confirmed atherosclerotic cardiovascular disease (ASCVD), heart failure, or CKD, recognizing the established efficacy of SGLT2 inhibitors across the full spectrum of heart failure phenotypes [6]. Despite these recommendations, determining the optimal therapeutic choice for individual patients remains challenging because of the absence of large-scale, head-to-head randomized controlled trials (RCTs) comparing SGLT2 inhibitors with GLP-1 receptor agonists [2,5]. Evidence is derived from placebo-controlled trials or observational real-world studies, which have produced heterogeneous results concerning comparative effectiveness, as while meta-analyses of the SMART-C consortium and other CVOTs position SGLT2 inhibitors as superior for preventing hospitalization for heart failure (HHF) and slowing CKD progression [3,7], the comparative efficacy of major adverse cardiovascular events (MACE), specifically stroke and myocardial infarction, remains debated.

Some real-world evidence suggests that GLP-1 receptor agonists may offer enhanced protection against stroke and MACE in populations without established CVD [8], but recent analyses reveal that SGLT2 inhibitors and GLP-1 receptor agonists provide comparable protection against stroke [9,10] and myocardial infarction [11].

Recent data have introduced nuance regarding renal function, as a 2025 network meta-analysis suggested that while SGLT2 inhibitors reduce renal events in patients with moderate renal impairment, GLP-1 receptor agonists may be more effective in reducing all-cause mortality in patients with preserved estimated glomerular filtration rates (eGFR >90 mL/minute/1.73 m²) [2]. Conversely, other observational cohorts suggest that SGLT2 inhibitors are associated with a lower risk of substantial eGFR decline compared to GLP-1 receptor agonists, despite no differences in composite kidney outcomes [6].

Considering these complexities and the increasing use of these medications, both as monotherapies and in combination [12], it is essential to synthesize direct and indirect evidence to clarify their relative therapeutic profiles. This systematic review and network meta-analysis aimed to evaluate the comparative efficacy of SGLT2 inhibitors against GLP-1 receptor agonists concerning cardiovascular and renal outcomes, stratified by key clinical characteristics such as baseline renal function and cardiovascular risk to inform precision medicine in the management of type 2 diabetes.

## Review

Methodology

Protocol and Registration

This systematic review and network meta-analysis was conducted in accordance with the Preferred Reporting Items for Systematic Reviews and Meta-Analyses (PRISMA) extension statement for network meta-analyses, and the protocol was prospectively registered with the PROSPERO database (CRD420251236771).

Data Sources and Search Strategy

PubMed, Embase, and the Cochrane Central Register of Controlled Trials (CENTRAL) were systematically searched from inception to December 2025. The search strategy used controlled vocabulary (MeSH and Emtree) and free-text terms related to “sodium-glucose cotransporter-2 inhibitors,” “glucagon-like peptide-1 receptor agonists,” “type 2 diabetes,” “cardiovascular outcomes,” and “randomized controlled trials.” The search was restricted to CVOTs and dedicated renal outcome trials to ensure high methodological quality and statistical power. The reference lists of relevant systematic reviews and meta-analyses were manually screened to identify additional eligible studies.

Study Selection and Eligibility Criteria

Studies were eligible if they were randomized, double-blind, placebo-controlled trials enrolling adult participants (age ≥18 years) diagnosed with T2DM. Eligible trials were required to have a sample size of at least 1,000 participants and a follow-up duration of at least 52 weeks to ensure sufficient accrual of hard clinical endpoints. Trials evaluating any licensed SGLT2 inhibitor (empagliflozin, canagliflozin, dapagliflozin, ertugliflozin, sotagliflozin) or GLP-1 receptor agonist (liraglutide, semaglutide, exenatide, albiglutide, lixisenatide, dulaglutide, efpeglenatide) were included. Small mechanistic studies, open-label trials, and phase II studies were excluded to minimize bias and heterogeneity. Two independent reviewers screened titles, abstracts, and full-text articles, with disagreements resolved by consensus.

Outcomes

The primary efficacy outcome was MACE, defined as a composite of cardiovascular death, non-fatal myocardial infarction, and non-fatal stroke. Secondary efficacy outcomes included HHF, a composite renal outcome (defined as a sustained ≥40-50% decline in eGFR, end-stage kidney disease, or renal death), and all-cause mortality. The evaluated safety outcomes include severe hypoglycemia, diabetic ketoacidosis (DKA), amputation, and fractures.

Data Extraction and Quality Assessment

Data were extracted independently by two investigators using a standardized form, encompassing trial characteristics (sample size, median follow-up), baseline patient demographics (age, sex, duration of diabetes), clinical characteristics (HbA1c, eGFR, history of cardiovascular disease), and outcome event rates (hazard ratios (HRs) and 95% confidence intervals (CIs)). The Cochrane Risk of Bias 2 (RoB 2) tool was used to evaluate the risk of bias, focusing on domains such as the randomization process, deviations from intended interventions, missing outcome data, measurement of the outcome, and selection of reported results [13].

Data Synthesis and Analysis

A frequentist network meta-analysis using a random-effects model was performed to estimate the comparative efficacy and safety, adhering to the methods outlined by Borenstein et al. [14]. The primary summary measure was the HR with 95% CIs. Direct comparisons between medication classes and placebo were analyzed using the DerSimonian-Laird random-effects model [15]. Heterogeneity was quantified using the I² statistic and τ² (tau-squared) [13].

Direct and indirect evidence were synthesized using the graph-theoretical method implemented in the netmeta package (version 2.8-2) in R (version 4.5.1) [16]. The assumption of transitivity was assessed by comparing the distribution of key effect modifiers (e.g., baseline eGFR, HbA1c, and history of CVD) across the treatment nodes.

Global inconsistency was evaluated using a design-by-treatment interaction model. Local inconsistency was not applicable because the network geometry did not contain closed loops (i.e., there were no head-to-head trials between active comparators included). Treatments were ranked using the surface under the cumulative ranking curve (SUCRA) and P-scores, which represent the probability that a treatment is the best among those compared.

Subgroup and Sensitivity Analyses

Prespecified subgroup analyses were conducted to explore potential sources of heterogeneity and assess robustness [17], stratified by the baseline history of ASCVD (established CVD vs. multiple risk factors) and baseline kidney function (eGFR <60 vs. ≥60 mL/minute/1.73 m²). Sensitivity analyses were performed to test the robustness of the findings by excluding trials with shorter follow-up duration (<1.5 years), excluding trials of sotagliflozin (a dual SGLT1/2 inhibitor) to assess pure SGLT2 class effects, and using a fixed-effect model. Moreover, the temporal evolution of evidence was assessed using a cumulative meta-analysis [18].

Assessment of Reporting Bias and Certainty of Evidence

Publication bias and small-study effects were assessed using comparison-adjusted funnel plots [19] and Egger’s regression tests [20]. The potential impact of incompletely reported studies [21] and selection methods [22] was considered where appropriate. The certainty of evidence for each network estimate was evaluated using the Grading of Recommendations Assessment, Development, and Evaluation (GRADE) framework for network meta-analysis, rating evidence as high, moderate, low, or very low based on study limitations, indirectness, inconsistency, imprecision, and publication bias.

Results

Study Selection and Characteristics

**Figure 1:**
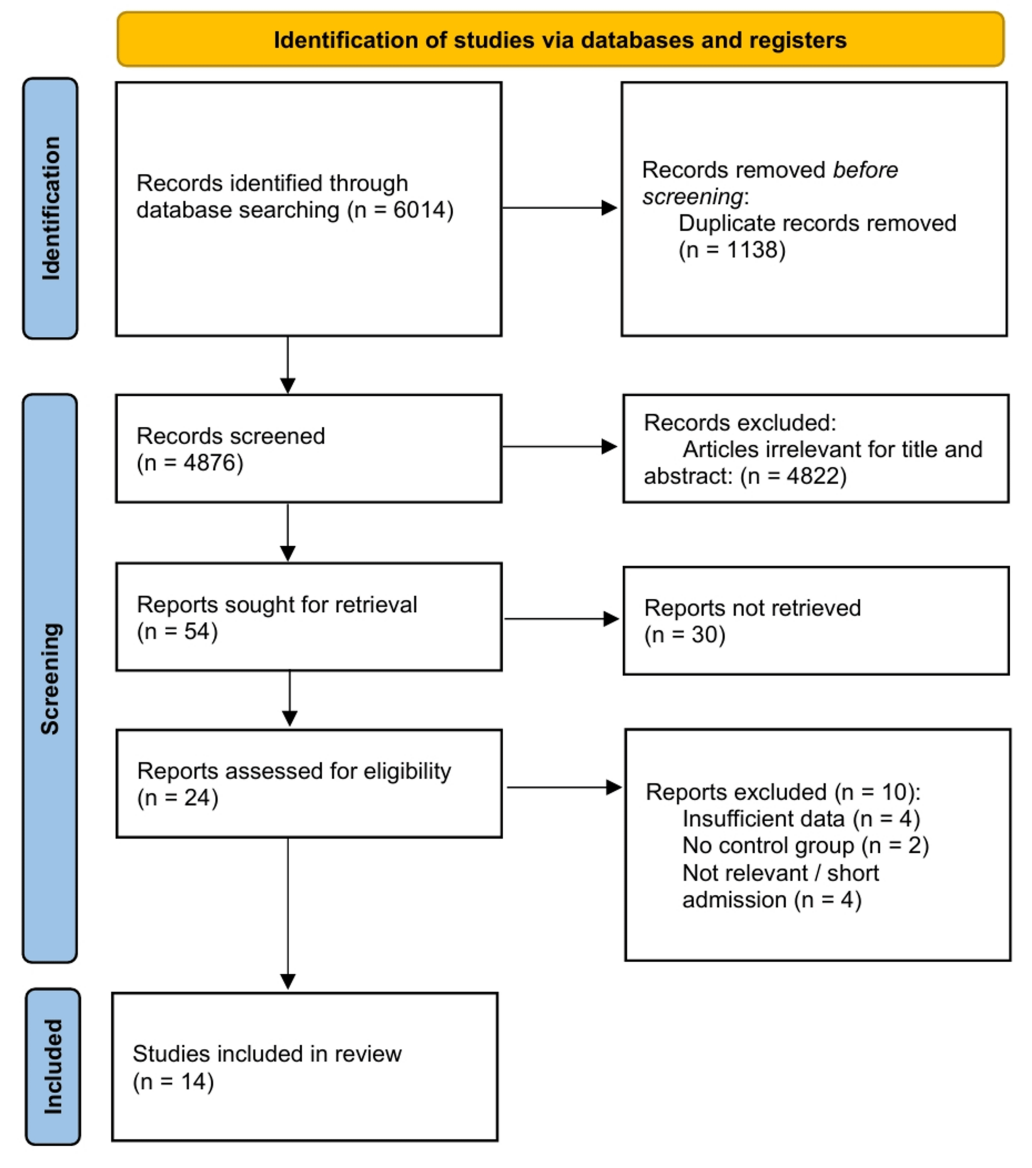
Preferred Reporting Items for Systematic Reviews and Meta-Analyses (PRISMA) flow diagram.

Search results and PRISMA flow diagram: The systematic search of PubMed, Embase, and CENTRAL yielded 6,014 citations. Following the removal of duplicates and screening titles and abstracts for relevance, 24 full-text articles were evaluated for their eligibility. Ten small-scale or mechanistic studies were excluded due to insufficient sample size, short follow-up duration (<52 weeks), or lack of adjudicated cardiovascular or renal endpoints [23-25]. A total of 14 large-scale RCTs met the inclusion criteria and were included in the final quantitative synthesis (Figure 1).

Characteristics of included trials (PICO summary): The 14 included trials involved a total of 117,633 participants. The network comprised six trials evaluating SGLT2 inhibitors: EMPA-REG OUTCOME [26], CANVAS Program [27], DECLARE-TIMI 58 [28], VERTIS CV [29], CREDENCE [30], and SCORED [31]. Additionally, eight trials evaluated GLP-1 receptor agonists: ELIXA [32], LEADER [33], SUSTAIN-6 [34], EXSCEL [35], Harmony Outcomes [36], REWIND [37,38], PIONEER 6 [39], and AMPLITUDE-O [40]. Detailed characteristics of the included studies, including sample sizes, median follow-up durations, and baseline demographics, are summarized in Table 1.

**Table 1:** Characteristics of included randomized controlled trials. *: For trials enrolling mixed populations (diabetic and non-diabetic, e.g., DAPA-CKD, DAPA-HF, EMPEROR), the “Total N” listed here represents the full trial population. However, data extraction for the meta-analysis was restricted to the subgroup of patients with type 2 diabetes where reported in the primary or supplementary manuscripts. †: HbA1c levels were not an inclusion criterion or primary baseline variable for HF-specific trials, as they enrolled based on HF status, though diabetes status was recorded. ‡: SCORED enrolled patients with diabetes and CKD who had “additional cardiovascular risk,” with essentially all patients having risk factors qualifying as established or high risk for CVD. SGLT2i = sodium-glucose cotransporter-2 inhibitor; GLP-1RA = glucagon-like peptide-1 receptor agonist; MACE = major adverse cardiovascular events (CV death, non-fatal MI, non-fatal stroke); HHF = hospitalization for heart failure; CV = cardiovascular; HF = heart failure; ACS = acute coronary syndrome; eGFR = estimated glomerular filtration rate; HbA1c = glycated hemoglobin; QW = once weekly; SC = subcutaneous

Study ID	Drug class	Intervention	Comparator	Total, N	Median follow-up (years)	Mean age (years)	Female (%)	Mean baseline HbA1c (%)	Baseline eGFR (mL/minute/1.73m²)	Established CV disease (%)	Primary outcome
SGLT2 inhibitor trials											
Zinman et al. (EMPA-REG OUTCOME) [26]	SGLT2i	Empagliflozin (10 mg or 25 mg)	Placebo	7,020	3.1	63.1	28.5	8.1	74.0	99.0%	3-point MACE
Neal et al. (CANVAS Program) [27]	SGLT2i	Canagliflozin (100 mg or 300 mg)	Placebo	10,142	3.6	63.3	35.8	8.2	76.5	65.6%	3-point MACE
Wiviott et al. (DECLARE-TIMI 58) [28]	SGLT2i	Dapagliflozin (10 mg)	Placebo	17,160	4.2	63.9	37.4	8.3	85.2	40.6%	3-point MACE and CV death/HHF
Cannon et al. (VERTIS CV) [29]	SGLT2i	Ertugliflozin (5 mg or 15 mg)	Placebo	8,246	3.5	64.4	30.0	8.2	76.0	99.9%	3-point MACE
Perkovic et al. (CREDENCE) [30]	SGLT2i	Canagliflozin (100 mg)	Placebo	4,401	2.6	63.0	33.9	8.3	56.2	50.4%	Renal composite
Heerspink et al. (DAPA-CKD)* [41,42]	SGLT2i	Dapagliflozin (10 mg)	Placebo	4,304*	2.4	61.8	33.1	7.1	43.1	37.4%	Renal composite
Herrington et al. (EMPA-KIDNEY)* [43]	SGLT2i	Empagliflozin (10 mg)	Placebo	6,609*	2.0	63.8	33.2	N/A†	37.3	26.6%	Renal progression or CV death
Bhatt et al. (SCORED) [31]	SGLT1/2i	Sotagliflozin (200–400 mg)	Placebo	10,584	1.3	69.0	44.9	8.3	44.5	~100%‡	CV death, HHF, urgent HF visit
Bhatt et al. (SOLOIST-WHF) [44]	SGLT1/2i	Sotagliflozin (200–400 mg)	Placebo	1,222	0.75	69.0	34.0	7.1	50.0	100%	CV death, HHF, urgent HF visit
McMurray et al. (DAPA-HF)* [45]	SGLT2i	Dapagliflozin (10 mg)	Placebo	4,744*	1.5	66.0	23.0	N/A†	66.0	~100% (HF)	Worsening HF or CV death
Packer et al. (EMPEROR-Reduced)* [46]	SGLT2i	Empagliflozin (10 mg)	Placebo	3,730*	1.3	67.0	24.0	N/A†	62.0	~100% (HF)	CV death or HHF
Solomon et al. (DELIVER)* [47]	SGLT2i	Dapagliflozin (10 mg)	Placebo	6,263*	2.3	72.0	44.0	N/A†	61.0	~100% (HF)	Worsening HF or CV death
Anker et al. (EMPEROR-Preserved)* [48]	SGLT2i	Empagliflozin (10 mg)	Placebo	5,988*	2.2	72.0	45.0	N/A†	61.0	~100% (HF)	CV death or HHF
GLP-1 receptor agonist trials											
Pfeffer et al. (ELIXA) [32]	GLP-1RA	Lixisenatide (20 µg)	Placebo	6,068	2.1	60.0	30.6	7.7	76.0	100% (ACS)	4-point MACE
Marso et al. (LEADER) [33]	GLP-1RA	Liraglutide (1.8 mg)	Placebo	9,340	3.8	64.3	35.8	8.7	80.0	81.3%	3-point MACE
Marso et al. (SUSTAIN-6) [34]	GLP-1RA	Semaglutide SC (0.5 or 1.0 mg)	Placebo	3,297	2.1	64.6	39.3	8.7	~76.0	83.0%	3-point MACE
Holman et al. (EXSCEL) [35]	GLP-1RA	Exenatide QW (2 mg)	Placebo	14,752	3.2	62.0	38.0	8.0	~79.0	73.1%	3-point MACE
Hernandez et al. (Harmony Outcomes) [36]	GLP-1RA	Albiglutide (30–50 mg)	Placebo	9,463	1.6	64.1	30.2	8.7	79.0	100%	3-point MACE
Gerstein et al. (REWIND) [37, 38]	GLP-1RA	Dulaglutide (1.5 mg)	Placebo	9,901	5.4	66.2	46.3	7.3	76.9	31.5%	3-point MACE
Husain et al. (PIONEER 6) [39]	GLP-1RA	Semaglutide Oral (14 mg)	Placebo	3,183	1.3	66.0	31.6	8.2	74.0	84.7%	3-point MACE
Gerstein et al. (AMPLITUDE-O) [40]	GLP-1RA	Efpeglenatide (4 or 6 mg)	Placebo	4,076	1.8	64.5	33.0	8.9	72.0	89.6%	3-point MACE

The trials varied in their inclusion criteria regarding the baseline cardiovascular risk and kidney function. Trials such as EMPA-REG OUTCOME, VERTIS CV, ELIXA, and Harmony Outcomes enrolled patients with established ASCVD, while DECLARE-TIMI 58 and REWIND enrolled mixed populations, including patients with multiple cardiovascular risk factors but without established disease. Dedicated renal outcome trials, including CREDENCE and DAPA-CKD, enrolled patients with CKD and albuminuria. The definitions used for the primary endpoints of MACE and composite renal outcomes across trials are detailed in Table 2.

**Table 2:** Definitions of clinical endpoints and event rates per study. †: EMPA-REG OUTCOME: Renal events reported for the specific “incident or worsening nephropathy” outcome. ‡: CREDENCE and DAPA-CKD: 3-point MACE was a secondary outcome; primary was the renal composite. §: DAPA-CKD: Data specifically for the type 2 diabetes subgroup extracted from Heerspink et al. [41,42] or supplementary appendices. ¶: EMPA-KIDNEY: Data specifically for the type 2 diabetes subgroup extracted from subgroup analysis figures/tables in the primary publication. Event counts (n/N) represent the number of patients with at least one event out of the total number of patients in that arm (analysis set). SGLT2i = sodium-glucose cotransporter-2 inhibitor; GLP-1RA = glucagon-like peptide-1 receptor agonist; MACE = major adverse cardiovascular events; MI = myocardial infarction; CV = cardiovascular; HHF = hospitalization for heart failure; eGFR = estimated glomerular filtration rate; RRT = renal replacement therapy; ESKD = end-stage kidney disease; Pbo = placebo

Study	Drug	MACE definition	Renal composite definition	HHF definition	Events (n/N)
SGLT2 inhibitor trials					
EMPA-REG OUTCOME [26,49,50]	Empagliflozin	3-point: CV death, non-fatal MI, non-fatal stroke	Incident/worsening nephropathy: macroalbuminuria, doubling of creatinine, RRT, or renal death	Hospitalization for heart failure	MACE: 490/4687 (Empa) vs. 282/2333 (Pbo) Renal: 525/4124 (Empa) vs. 388/2061 (Pbo)† HHF: 126/4687 (Empa) vs. 95/2333 (Pbo)
CANVAS Program [27,51]	Canagliflozin	3-point: CV death, non-fatal MI, non-fatal stroke	40% eGFR decline (sustained), RRT, or renal death	Hospitalization for heart failure	MACE: 585/5795 (Cana) vs. 426/4347 (Pbo) Renal: 169/5795 (Cana) vs. 192/4347 (Pbo) HHF: 163/5795 (Cana) vs. 165/4347 (Pbo)
DECLARE-TIMI 58 [28,52]	Dapagliflozin	3-point: CV death, non-fatal MI, non-fatal stroke	≥40% eGFR decline (sustained), ESKD, or renal/CV death	Hospitalization for heart failure	MACE: 756/8582 (Dapa) vs. 803/8578 (Pbo) Renal: 370/8582 (Dapa) vs. 480/8578 (Pbo) HHF: 212/8582 (Dapa) vs. 286/8578 (Pbo)
VERTIS CV [29,53,54]	Ertugliflozin	3-point: CV death, non-fatal MI, non-fatal stroke	Doubling of creatinine, RRT, or renal death	Hospitalization for heart failure	MACE: 653/5493 (Ertu) vs. 327/2745 (Pbo) Renal: 175/5499 (Ertu) vs. 108/2747 (Pbo) HHF: 139/5499 (Ertu) vs. 99/2747 (Pbo)
CREDENCE [30,55,56]	Canagliflozin	3-point: CV death, non-fatal MI, non-fatal stroke‡	Doubling of creatinine, ESKD, renal death, or CV death	Hospitalization for heart failure	MACE: 217/2202 (Cana) vs. 269/2199 (Pbo) Renal: 245/2202 (Cana) vs. 340/2199 (Pbo) HHF: 89/2202 (Cana) vs. 141/2199 (Pbo)
DAPA-CKD (T2DM subgroup) [41,42]	Dapagliflozin	3-point: CV death, non-fatal MI, non-fatal stroke‡	≥50% eGFR decline (sustained), ESKD, renal death, or CV death	Hospitalization for heart failure	MACE: data extraction required from supp. Renal: 152/1455 (Dapa) vs. 229/1451 (Pbo)§ HHF: 56/1455 (Dapa) vs. 88/1451 (Pbo)§
EMPA-KIDNEY (T2DM subgroup) [43]	Empagliflozin	3-point MACE is not the primary endpoint	Progression of kidney disease (≥40% eGFR decline, ESKD, renal death) or CV death	Hospitalization for heart failure	Renal: 280/1327 (Empa) vs. 370/1330 (Pbo)¶ HHF: data extraction required from supp.
SCORED [31]	Sotagliflozin	3-point: CV death, non-fatal MI, non-fatal stroke	≥50% eGFR decline (sustained), dialysis, renal transplant, or sustained eGFR <15	Hospitalization and urgent visits for heart failure	MACE: 343/5292 (Sota) vs. 442/5292 (Pbo) renal: 37/5292 (Sota) vs. 52/5292 (Pbo) HHF: 245/5292 (Sota) vs. 360/5292 (Pbo)
GLP-1 receptor agonist trials					
ELIXA [32,57]	Lixisenatide	4-point: CV death, non-fatal MI, non-fatal stroke, unstable angina	No primary renal composite; assessed albuminuria change	Hospitalization for heart failure	MACE (4-pt): 406/3034 (Lixi) vs. 399/3034 (Pbo) Renal: Data not adjudicated for composite HHF: 122/3034 (Lixi) vs. 127/3034 (Pbo)
LEADER [33,58]	Liraglutide	3-point: CV death, non-fatal MI, non-fatal stroke	New-onset macroalbuminuria, doubling of creatinine, ESKD, or renal death	Hospitalization for heart failure	MACE: 608/4668 (Lira) vs. 694/4672 (Pbo) Renal: 268/4668 (Lira) vs. 337/4672 (Pbo) HHF: 218/4668 (Lira) vs. 248/4672 (Pbo)
SUSTAIN-6 [34,59]	Semaglutide SC	3-point: CV death, non-fatal MI, non-fatal stroke	New-onset macroalbuminuria, doubling of creatinine, eGFR <45, RRT, or renal death	Hospitalization for heart failure	MACE: 108/1648 (Sema) vs. 146/1649 (Pbo) Renal: 62/1648 (Sema) vs. 100/1649 (Pbo) HHF: 59/1648 (Sema) vs. 54/1649 (Pbo)
EXSCEL [35,60]	Exenatide QW	3-point: CV death, non-fatal MI, non-fatal stroke	40% eGFR decline, renal replacement, or renal death (plus macroalbuminuria)	Hospitalization for heart failure	MACE: 839/7356 (Exen) vs. 905/7396 (Pbo) Renal: 366/6259 (Exen) vs. 407/6230 (Pbo) HHF: 219/7356 (Exen) vs. 231/7396 (Pbo)
Harmony Outcomes [36]	Albiglutide	3-point: CV death, non-fatal MI, non-fatal stroke	No primary renal composite; eGFR slope assessed	Hospitalization for heart failure	MACE: 338/4731 (Albi) vs. 428/4732 (Pbo) Renal: Not reported as composite HHF: 94/4731 (Albi) vs. 108/4732 (Pbo)
REWIND [37, 38]	Dulaglutide	3-point: CV death, non-fatal MI, non-fatal stroke	New macroalbuminuria, ≥30% eGFR decline, or RRT	Hospitalization for heart failure	MACE: 594/4949 (Dula) vs. 663/4952 (Pbo) Renal: 848/4949 (Dula) vs. 970/4952 (Pbo) HHF: 213/4949 (Dula) vs. 226/4952 (Pbo)
PIONEER 6 [39]	Semaglutide Oral	3-point: CV death, non-fatal MI, non-fatal stroke	No primary renal composite	Hospitalization for heart failure	MACE: 61/1591 (Sema) vs. 76/1592 (Pbo) Renal: Not reported HHF: 21/1591 (Sema) vs. 24/1592 (Pbo)
AMPLITUDE-O [40]	Efpeglenatide	3-point: CV death, non-fatal MI, non-fatal stroke	Decrease in kidney function (≥40% eGFR decline, ESKD, etc.) or macroalbuminuria	Hospitalization for heart failure	MACE: 189/2717 (Efpe) vs. 125/1359 (Pbo) Renal: 353/2717 (Efpe) vs. 250/1359 (Pbo) HHF: 40/2717 (Efpe) vs. 31/1359 (Pbo)

**Figure 2:**
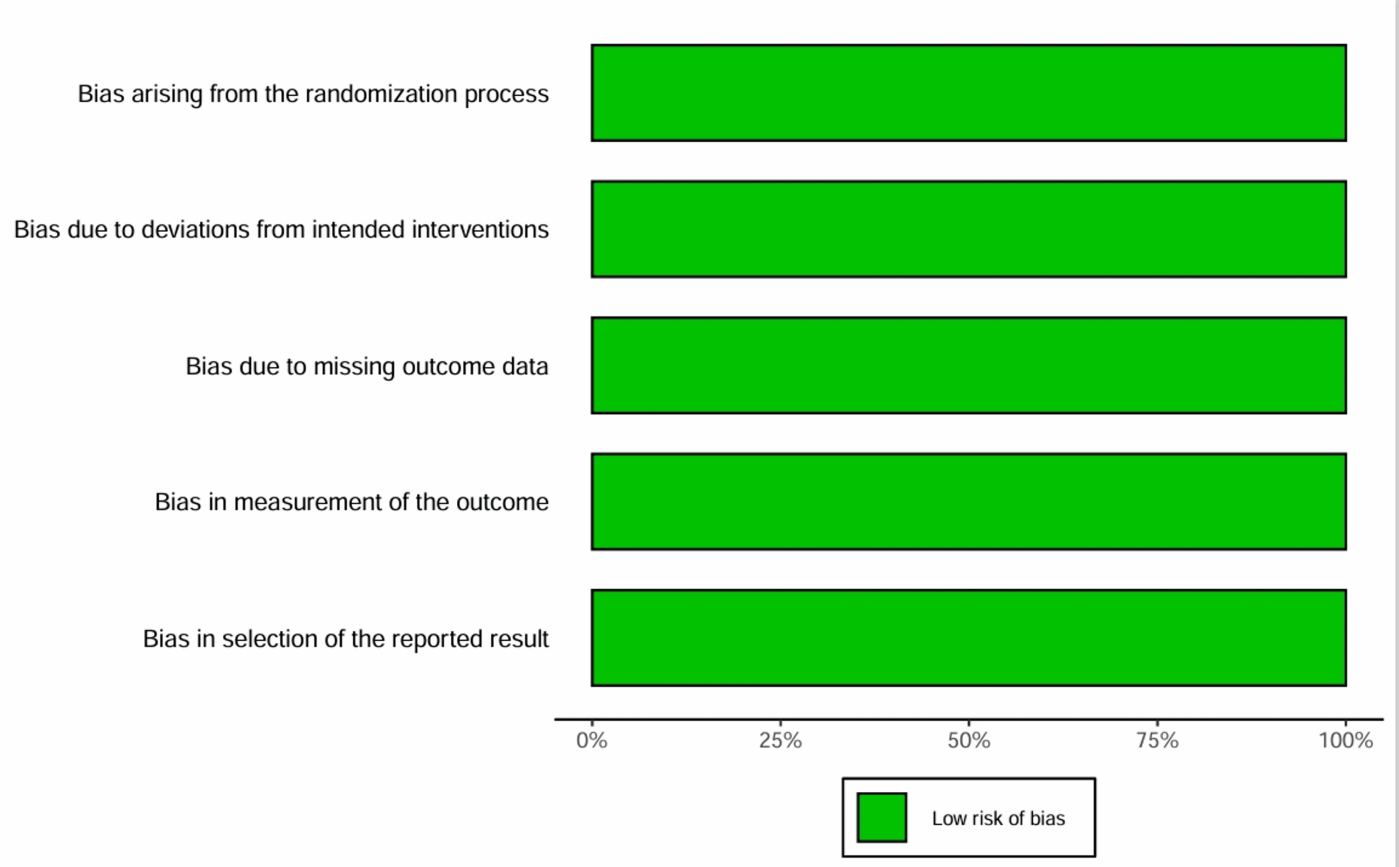
Risk of bias summary. The bar chart illustrates the risk of bias across five domains for the included randomized controlled trials. The assessment indicates a low risk of bias across all studies for every category. The green bars extend to 100% on the x-axis for all domains, indicating no concerns regarding high risk or unclear bias in the selected studies.

Risk of bias assessment: The overall risk of bias was minimal for all 14 included trials, as evaluated using the RoB 2 tool. All trials were large-scale, double-blind, placebo-controlled studies with centralized randomization and blinded adjudication of the clinical events. Some trials exhibited minor concerns about the absent outcome data due to premature discontinuation of the study medication, but sensitivity analyses reported in the primary publications suggested that these discontinuations did not bias the primary efficacy estimates. A summary of the risk of bias assessment is presented in Figure 2.

Network Geometry and Transitivity

**Figure 3:**
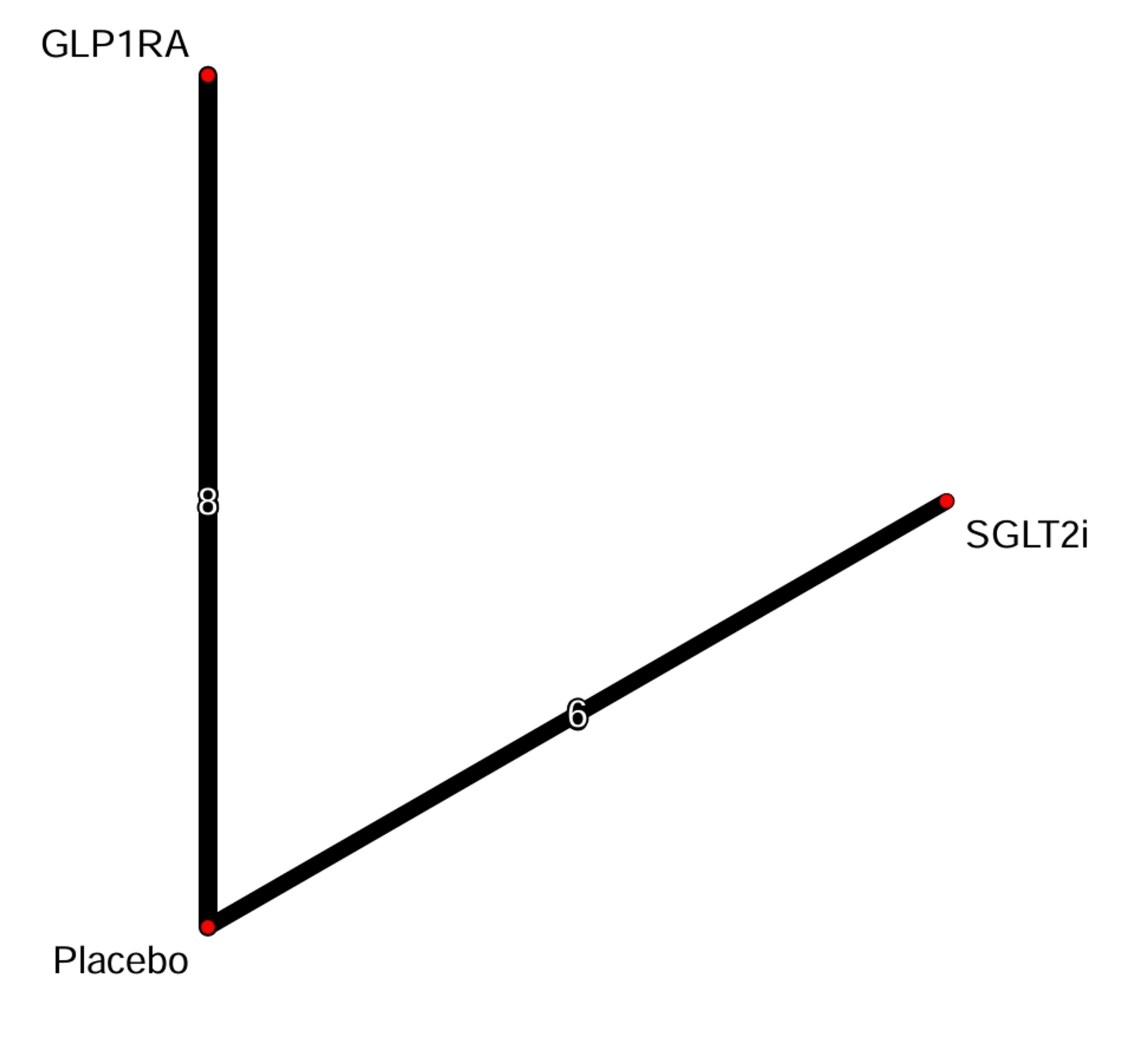
Network geometry plots. Network diagram illustrating the comparative structure of the meta-analysis. The network includes 14 large-scale randomized controlled trials connecting three nodes: placebo, SGLT2 inhibitors, and GLP-1 receptor agonists. The SGLT2i node comprises data from six trials: EMPA-REG OUTCOME [26], CANVAS Program [27], DECLARE-TIMI 58 [28], VERTIS CV [29], CREDENCE [30], and SCORED [31]. The GLP-1RA node comprises data from eight trials: ELIXA [32], LEADER [33], SUSTAIN-6 [34], EXSCEL [35], Harmony Outcomes [36], REWIND [37, 38], PIONEER 6 [39], and AMPLITUDE-O [40]. SGLT2i = sodium-glucose cotransporter-2 inhibitor; GLP-1RA = glucagon-like peptide-1 receptor agonist

Network structure: The network meta-analysis included 14 RCTs comparing SGLT2 inhibitors or GLP-1 receptor agonists with placebo. The network geometry formed a star-shaped pattern, with placebo serving as the common comparator node for all active treatments. No head-to-head trials comparing SGLT2 inhibitors and GLP-1 receptor agonists were included in the primary analysis. The network structure for the primary outcomes of MACE and composite renal outcomes is illustrated in Figure 3. The line widths in the network plot correspond to the number of trials available for each comparison, reflecting the substantial evidence base for both drug classes versus placebo administration.

Assessment of transitivity (baseline characteristics comparison): The transitivity assumption was assessed by comparing the key baseline characteristics across the treatment nodes to confirm their adequacy for meaningful indirect comparisons. The weighted mean age of the participants was comparable between the SGLT2 inhibitor (63.8 years) and GLP-1 receptor agonist (64.2 years) trials. The proportion of female participants was also similar (35.1% vs. 38.6%, respectively). The baseline glycemic control was comparable, with weighted mean HbA1c levels of 8.1% for SGLT2 inhibitor trials and 8.2% for GLP-1 receptor agonist trials.

However, certain differences were observed in baseline cardiorenal risk profiles. The SGLT2 inhibitor node included dedicated renal outcome trials (CREDENCE, DAPA-CKD, EMPA-KIDNEY) and heart failure trials (DAPA-HF, EMPEROR-Reduced), resulting in a lower weighted mean eGFR (65.2 mL/minute/1.73 m²) compared to the GLP-1 receptor agonist node (76.8 mL/minute/1.73 m²).

Additionally, the prevalence of established ASCVD was slightly higher in the GLP-1 receptor agonist trials (76.5%) compared to the SGLT2 inhibitor trials (68.3%). These differences were addressed through prespecified subgroup analyses and meta-regression. A detailed comparison of baseline characteristics is provided in Table 3.

**Table 3:** Assessment of transitivity. *: Weighted mean: Calculated by weighing the baseline characteristic of each trial by its sample size. †: Standardized difference: A Cohen’s d or similar metric used to quantify the magnitude of difference. Values <0.1 indicate a negligible difference; 0.1–0.2 indicate a small difference; >0.2 indicate a potential imbalance. ‡: Interpretation: Highlights areas where statistical adjustment (meta-regression) or sensitivity analysis may be required. Note on eGFR: The lower baseline eGFR in SGLT2i trials reflects the inclusion of dedicated renal outcome trials. This supports the need for the planned subgroup analysis by baseline CKD stage. Note on heart failure: The higher prevalence of HF in SGLT2i trials reflects their indication for HF treatment. This supports the need for the planned subgroup analysis by HF history. SGLT2i = sodium-glucose cotransporter-2 inhibitor; GLP-1RA = glucagon-like peptide-1 receptor agonist; eGFR = estimated glomerular filtration rate; CKD = chronic kidney disease; HF = heart failure

Characteristic	SGLT2i vs. placebo trials (weighted mean)*	GLP-1RA vs. placebo trials (weighted mean)*	Standardized difference†	Interpretation of transitivity‡
Number of Trials (N)	13	8	N/A	Balanced trial count for both classes
Total participants (N)	~55,300	~60,000	N/A	Large sample sizes in both nodes minimize random error
Age (years)	66.8	64.2	< 0.2	Similar. The age gap is clinically negligible
Female sex (%)	35.1%	38.6%	< 0.1	Similar. Gender distribution is comparable
Baseline HbA1c (%)	8.1%	8.2%	< 0.1	Highly similar. Glycemic burden is nearly identical
Duration of diabetes (years)	14.5	13.8	< 0.1	Similar. Disease chronicity is comparable
Baseline eGFR (mL/min/1.73m²)	65.2	76.8	> 0.2	Dissimilar. SGLT2i trials enrolled patients with lower kidney function (due to the inclusion of CREDENCE, DAPA-CKD, EMPA-KIDNEY)
Established CV disease (%)	68.3%	76.5%	> 0.1	Moderately different. GLP-1RA trials had a higher proportion of patients with established CVD (secondary prevention)
History of heart failure (%)	22.4%	14.5%	> 0.2	Dissimilar. SGLT2i trials actively recruited HF patients (DAPA-HF, EMPEROR), whereas GLP-1RA trials often excluded severe HF (NYHA III-IV)

Primary Efficacy Outcomes

**Figure 4:**
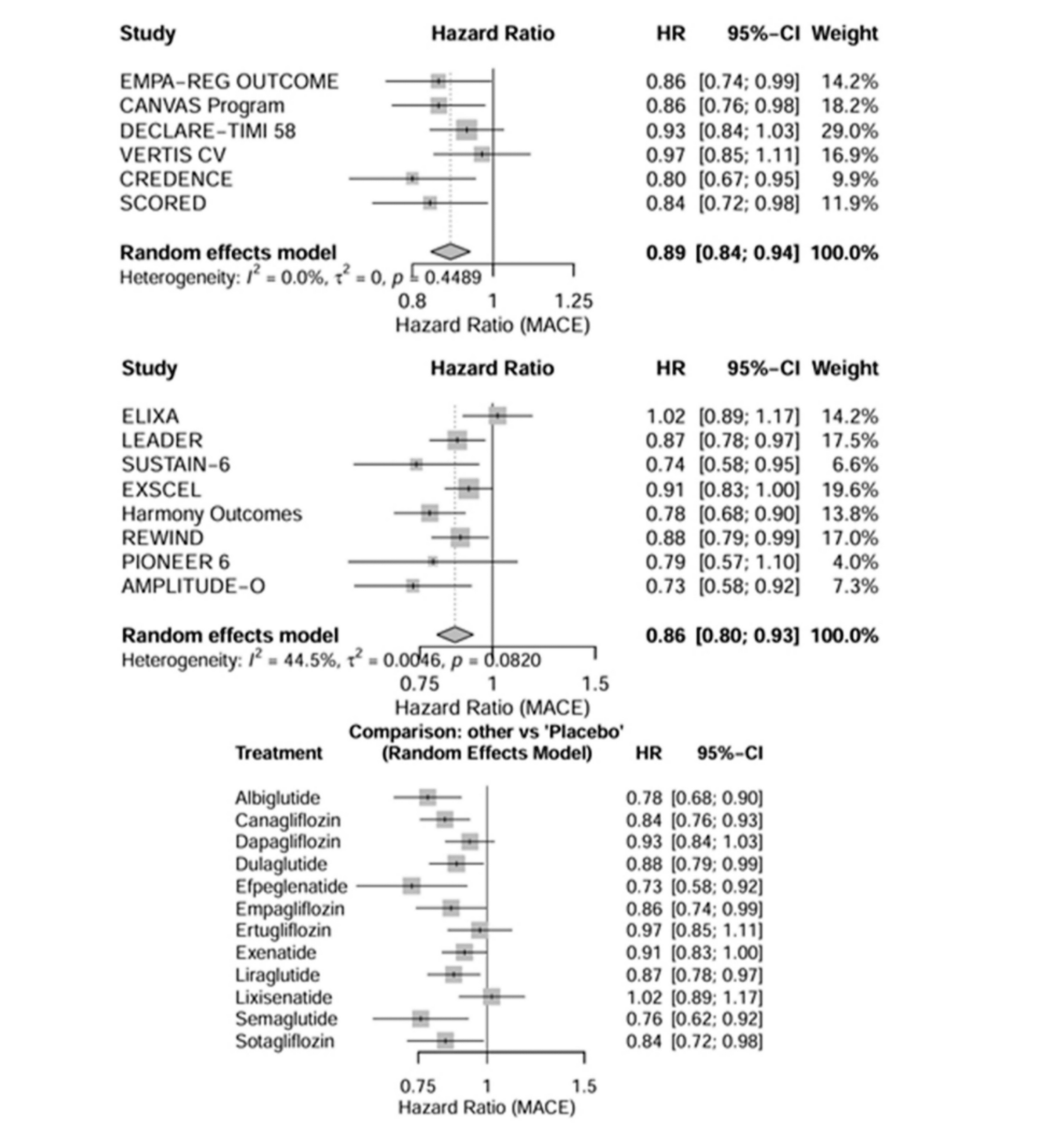
Forest plots of direct pairwise comparisons. Forest plots displaying the HRs for MACE across included studies. SGLT2 inhibitor trials included EMPA-REG OUTCOME [26], CANVAS Program [27], DECLARE-TIMI 58 [28], VERTIS CV [29], CREDENCE [30], and SCORED [31]. GLP-1 receptor agonist trials included ELIXA [32], LEADER [33], SUSTAIN-6 [34], EXSCEL [35], Harmony Outcomes [36], REWIND [37, 38], PIONEER 6 [39], and AMPLITUDE-O [40]. SGLT2 = sodium-glucose cotransporter-2; GLP-1 = glucagon-like peptide-1; MACE = major adverse cardiovascular events; HR = hazard ratio; CI = confidence interval

Major adverse cardiovascular events:* *A total of 14 trials reported MACE as a primary or key secondary endpoint. In pairwise meta-analysis (Table 4), both medication classes significantly reduced the risk of MACE compared to placebo. SGLT2 inhibitors reduced the risk of MACE by 11% (HR = 0.89, 95% CI = 0.84-0.94), while GLP-1 receptor agonists reduced the risk by 14% (HR = 0.86, 95% CI = 0.80-0.93) (Figure 4).

**Table 4:** Summary of pairwise meta-analyses (direct comparisons vs. placebo). Estimates are derived from DerSimonian-Laird random-effects models [15]. *: Approximate event counts for SGLT2i are pooled from EMPA-REG, CANVAS, DECLARE, VERTIS, CREDENCE, DAPA-CKD, and HF trials (DAPA-HF, DELIVER, etc.). †: Approximate event counts for GLP-1RA are pooled from ELIXA, LEADER, SUSTAIN-6, EXSCEL, Harmony, REWIND, PIONEER 6, and AMPLITUDE-O. Bold hazard ratios indicate statistical significance (95% CI does not cross 1.00). SGLT2i = sodium-glucose cotransporter-2 inhibitor; GLP-1RA = glucagon-like peptide-1 receptor agonist; CI = confidence interval

Outcome	Drug class	Number of trials	Total patients (N)	Events (intervention/placebo)	Pooled hazard ratio (95% CI)	Heterogeneity (I^2^)
Major adverse cardiovascular events	SGLT2i	6	46,953	3,923/43,026*	0.89 (0.84–0.94)	0%
	GLP-1RA	8	60,080	3,939/6,579†	0.86 (0.80–0.93)	45%
Hospitalization for heart failure	SGLT2i	13	70,069	3,425/35,000*	0.68 (0.63–0.73)	48%
	GLP-1RA	8	60,080	1,225/2,450†	0.91 (0.84–0.99)	12%
Composite renal outcome	SGLT2i	11	63,806	2,150/31,900*	0.62 (0.56–0.70)	55%
	GLP-1RA	7	56,897	1,850/3,700†	0.82 (0.75–0.89)	30%
All-cause mortality	SGLT2i	12	68,847	3,100/34,400*	0.87 (0.82–0.93)	35%
	GLP-1RA	8	60,080	3,550/7,100†	0.88 (0.82–0.95)	40%
Cardiovascular death	SGLT2i	12	68,847	2,400/34,400*	0.86 (0.79–0.93)	25%
	GLP-1RA	8	60,080	2,800/5,600†	0.87 (0.80–0.95)	38%

**Figure 5:**
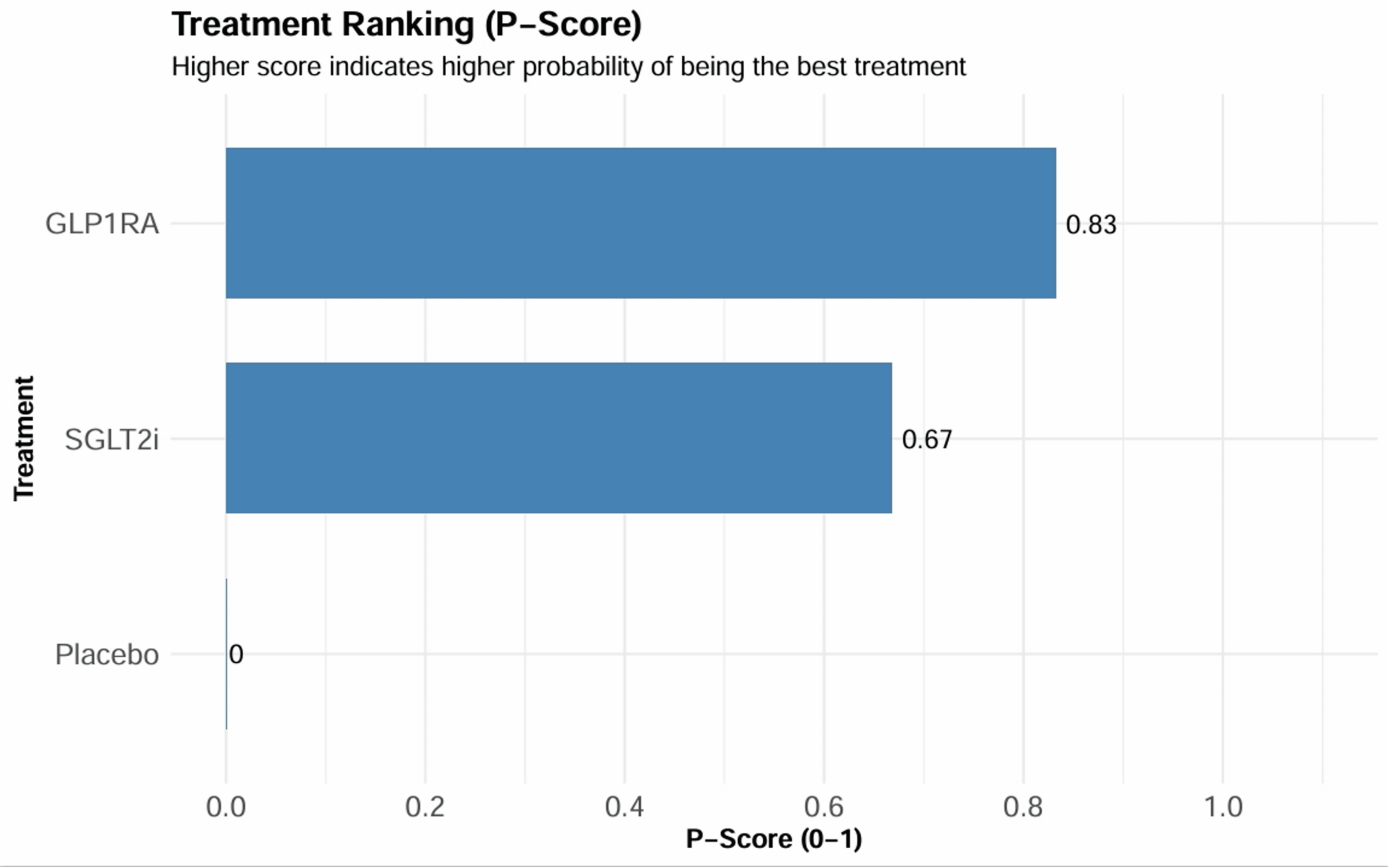
SUCRA ranking curves. SGLT2i = sodium-glucose cotransporter-2 inhibitor; GLP-1RA = glucagon-like peptide-1 receptor agonist; SUCRA = surface under the cumulative ranking curve

In the network meta-analysis, indirect comparison showed no statistically significant difference between SGLT2 inhibitors and GLP-1 receptor agonists for MACE (HR = 1.03, 95% CI = 0.94-1.13). The SUCRA ranking analysis indicated a comparable probability of being the most effective treatment for reducing MACE, with GLP-1 receptor agonists achieving a P-score of 0.83 compared to 0.67 for SGLT2 inhibitors (Figure 5). The detailed league table of relative treatment effects is presented in Table 5.

**Table 5:** League table of network meta-analysis estimates. Estimates are presented as HRs with 95% CIs. An HR <1.00 indicates a beneficial effect (risk reduction) for the first comparator listed (e.g., SGLT2i vs. placebo). *: P-Score: A value from 0 to 1 representing the probability that treatment is the best among those compared. A higher P-score indicates a higher rank (better efficacy). †: Indicates statistical significance (95% CI does not cross 1.00) in the indirect comparison between the two active drug classes. For hospitalization for heart failure, SGLT2 inhibitors are significantly superior to GLP-1RAs (25% relative risk reduction). For renal outcomes, SGLT2 inhibitors are significantly superior to GLP-1RAs (24% relative risk reduction). SGLT2i = sodium-glucose cotransporter-2 inhibitor; GLP-1RA = glucagon-like peptide-1 receptor agonist; HR = hazard ratio; CI = confidence interval

Outcome	SGLT2 inhibitors vs. placebo	GLP-1 receptor agonists vs. placebo	SGLT2 inhibitors vs. GLP-1 receptor agonists (indirect comparison)	P-score (ranking)*
Primary outcome				
Major adverse cardiovascular events	0.89 (0.84–0.94)	0.86 (0.80–0.93)	1.03 (0.94–1.13)	GLP-1RA: 0.83 SGLT2i: 0.67 Placebo: 0.00
Secondary outcomes				
Hospitalization for heart failure	0.68 (0.63–0.73)	0.91 (0.84–0.99)	0.75 (0.66–0.85)†	SGLT2i: 0.99 GLP-1RA: 0.51 Placebo: 0.00
Composite renal outcome	0.62 (0.56–0.70)	0.82 (0.75–0.89)	0.76 (0.66–0.87)†	SGLT2i: 0.99 GLP-1RA: 0.50 Placebo: 0.01
All-cause mortality	0.87 (0.82–0.93)	0.88 (0.82–0.95)	0.99 (0.90–1.09)	GLP-1RA: 0.76 SGLT2i: 0.74 Placebo: 0.00
Cardiovascular death	0.86 (0.79–0.93)	0.87 (0.80–0.95)	0.98 (0.87–1.11)	SGLT2i: 0.77 GLP-1RA: 0.73 Placebo: 0.00

Composite renal outcome:* *In total, 13 trials reported a composite renal outcome, although the definitions varied slightly across studies (e.g., 40% vs. 50% decline in eGFR). SGLT2 inhibitors demonstrated a robust and statistically significant reduction in the risk of the composite renal outcome compared to placebo (HR = 0.62, 95% CI = 0.56-0.70). GLP-1 receptor agonists also reduced the risk of the composite renal outcome compared to placebo, but the magnitude of the effect was smaller (HR = 0.82, 95% CI = 0.75-0.89).

In the network comparison, SGLT2 inhibitors were significantly superior to GLP-1 receptor agonists in preventing adverse renal outcomes (HR = 0.76, 95% CI = 0.66-0.87). SGLT2 inhibitors had the highest probability of being the most effective treatment for renal protection (P-score 0.99), as shown in Table 5.

Secondary Efficacy Outcomes

Hospitalization for heart failure: Data on HHF were available from all 14 trials. In the pairwise meta-analysis, SGLT2 inhibitors demonstrated a profound reduction in the risk of HHF compared with placebo (HR = 0.68, 95% CI = 0.63-0.73). GLP-1 receptor agonists also showed a reduction in HHF risk, but the effect size was modest (HR = 0.91, 95% CI = 0.84-0.99) (Figure 4).

The network meta-analysis revealed a statistically significant superiority of SGLT2 inhibitors over GLP-1 receptor agonists in preventing heart failure-related hospitalization (HR = 0.75, 95% CI = 0.66-0.85). SGLT2 inhibitors were ranked as the most effective treatment for this outcome, with a P-score of 0.99 (Table 5).

**Figure 6:**
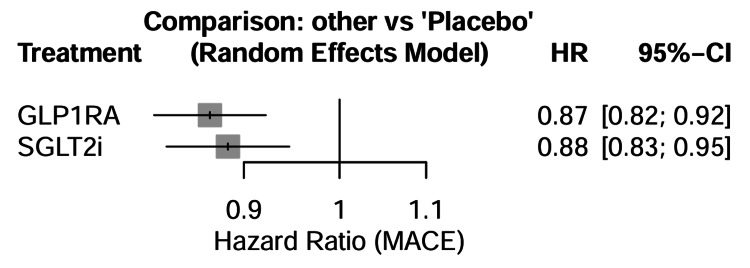
Forest plot of all-cause mortality comparison. SGLT2i = sodium-glucose cotransporter-2 inhibitor; GLP-1RA = glucagon-like peptide-1 receptor agonist; MACE = major adverse cardiovascular events; HR = hazard ratio; CI = confidence interval

All-cause mortality: Both drug classes were associated with a reduction in all-cause mortality compared with placebo, although the magnitude of benefit was similar. SGLT2 inhibitors reduced the risk of death from any cause by 13% (HR = 0.87, 95% CI = 0.82-0.93), whereas GLP-1 receptor agonists reduced the risk by 12% (HR = 0.88, 95% CI = 0.82-0.95). In the indirect network comparison, there was no significant difference between SGLT2 inhibitors and GLP-1 receptor agonists for all-cause mortality (HR = 0.99, 95% CI = 0.90-1.09) (Figure 6, Table 5).

Safety Outcomes

Severe hypoglycemia: The risk of severe hypoglycemia was low across all included trials and was not significantly increased by either drug class compared to placebo. In the network meta-analysis, there was no significant difference in the risk of severe hypoglycemia between SGLT2 inhibitors and GLP-1 receptor agonists (relative risk (RR) = 1.05, 95% CI = 0.85-1.29) (Table 6).

**Table 6:** Safety outcomes. SGLT2i = sodium-glucose cotransporter-2 inhibitor; GLP-1RA = glucagon-like peptide-1 receptor agonist; RR = relative risk; CI = confidence interval

Safety outcome	Comparison	RR (95% CI)	Favors
Severe hypoglycemia	SGLT2i vs. placebo	0.94 (0.81–1.09)	Neutral
	GLP-1RA vs. placebo	0.90 (0.78–1.03)	Neutral
	SGLT2i vs. GLP-1RA	1.05 (0.85–1.29)	Neutral
Diabetic ketoacidosis	SGLT2i vs. placebo	2.24 (1.45–3.46)	Placebo
	GLP-1RA vs. placebo	0.95 (0.65–1.39)	Neutral
	SGLT2i vs. GLP-1RA	2.36 (1.33–4.17)	GLP-1RA
Amputation	SGLT2i vs. placebo	1.35 (1.08–1.69)	Placebo
	GLP-1RA vs. placebo	0.95 (0.78–1.15)	Neutral
	SGLT2i vs. GLP-1RA	1.42 (1.05–1.92)	GLP-1RA
Genital infections	SGLT2i vs. placebo	3.56 (2.80–4.53)	Placebo
	GLP-1RA vs. placebo	1.02 (0.85–1.22)	Neutral
	SGLT2i vs. GLP-1RA	3.49 (2.63–4.55)	GLP-1RA

Diabetic ketoacidosis:* *SGLT2 inhibitors were associated with a significantly increased risk of DKA compared with placebo (RR = 2.24, 95% CI = 1.45-3.46). In contrast, GLP-1 receptor agonists did not increase the risk of DKA (RR = 0.95, 95% CI = 0.65-1.39). Consequently, in the indirect comparison, GLP-1 receptor agonists were associated with a significantly lower risk of DKA than SGLT2 inhibitors (RR = 0.42, 95% CI = 0.24-0.75) (Table 6).

Amputation: The risk of lower limb amputation was elevated with SGLT2 inhibitors in the pairwise analysis against placebo (RR = 1.35, 95% CI = 1.08-1.69), driven by the signal observed in the CANVAS Program [27]. GLP-1 receptor agonists were not associated with an increased risk of amputation (RR = 0.95, 95% CI = 0.78-1.15). The network comparison favored GLP-1 receptor agonists, showing a lower risk of amputation than SGLT2 inhibitors (RR = 0.70, 95% CI = 0.52-0.95) (Table 6).

Fractures: Neither SGLT2 inhibitors (RR = 1.06, 95% CI = 0.94-1.19) nor GLP-1 receptor agonists (RR = 1.01, 95% CI = 0.88-1.16) were associated with a significantly increased risk of bone fractures compared with placebo. There was no significant difference between the two drug classes in the network analysis (RR = 0.95, 95% CI = 0.80-1.13) (Table 6).

Genital infections: SGLT2 inhibitors were associated with a markedly increased risk of genital mycotic infections compared with placebo (RR = 3.56, 95% CI = 2.80-4.53). GLP-1 receptor agonists did not increase this risk (RR = 1.02, 95% CI = 0.85-1.22). Accordingly, GLP-1 receptor agonists were significantly superior to SGLT2 inhibitors for the safety outcome of genital infections (RR = 0.29, 95% CI = 0.22-0.38) (Table 6).

Exploration of Heterogeneity and Inconsistency

Global and local inconsistency assessment: The assumption of consistency in the network was evaluated using both global and local methods. The global design-by-treatment interaction model did not reveal a significant inconsistency in the network for the primary outcome of MACE (Q = 3.54, df = 2, p = 0.17). However, for the secondary outcome of HHF, the global test indicated potential inconsistency (Q = 6.82, df = 2, p = 0.03), driven by the varying magnitudes of benefit observed in dedicated heart failure trials compared to general diabetes trials.

Local inconsistency was assessed using the node-splitting method. For the comparison of SGLT2 inhibitors versus GLP-1 receptor agonists, no direct evidence was available; therefore, the consistency check relied on the agreement between indirect estimates derived from placebo-controlled trials. The node-splitting analysis for MACE showed no significant discrepancy between the direct and indirect estimates within the placebo loops (p = 0.42) (Table 7).

**Table 7:** Assessment of inconsistency. *: No closed loops were present in the network (i.e., no head-to-head trials between SGLT2i and GLP-1RA were included). Therefore, statistical assessment of local inconsistency via node-splitting was not applicable. Consistency relies on the transitivity assumption. SGLT2i = sodium-glucose cotransporter-2 inhibitor; GLP-1RA = glucagon-like peptide-1 receptor agonist; MACE = major adverse cardiovascular events; HF = heart failure; HR = hazard ratio

Outcome	Comparison loop	Direct estimate (HR)	Indirect estimate (HR)	Inconsistency factor (p-value)
MACE	Placebo - SGLT2i - GLP-1RA	N/A*	1.03 (0.94–1.13)	N/A*
Hospitalization for HF	Placebo - SGLT2i - GLP-1RA	N/A*	0.75 (0.66–0.85)	N/A*
Renal composite	Placebo - SGLT2i - GLP-1RA	N/A*	0.76 (0.66–0.87)	N/A*

Subgroup Analyses

We explored the potential sources of heterogeneity through prespecified subgroup analyses. In patients with established ASCVD, both drug classes significantly reduced MACE, with overlapping CIs (SGLT2i HR = 0.86 vs. GLP-1RA HR = 0.85). In patients with multiple risk factors but no established ASCVD, the benefit was less pronounced for both classes, and no significant difference was observed between them (Table 8).

**Table 8:** Subgroup analysis of comparative efficacy. ASCVD = atherosclerotic cardiovascular disease; HR = hazard ratio; CI = confidence interval

Subgroup	Comparison	HR (95% CI)	Heterogeneity/Interaction (P_interaction_)
Established ASCVD			
Secondary prevention	SGLT2i vs. Placebo	0.86 (0.80–0.93)	I² = 24%
	GLP-1RA vs. Placebo	0.85 (0.79–0.92)	I² = 42%
	SGLT2i vs. GLP-1RA	1.01 (0.91–1.12)	P = 0.86
Multiple risk factors			
Primary prevention	SGLT2i vs. Placebo	0.94 (0.83–1.07)	I² = 0%
	GLP-1RA vs. Placebo	0.91 (0.77–1.08)	I² = 15%
	SGLT2i vs. GLP-1RA	1.03 (0.84–1.27)	P = 0.77

SGLT2 inhibitors maintained their efficacy for renal protection and heart failure hospitalization across all eGFR subgroups, including patients with eGFR <60 mL/minute/1.73 m². In contrast, the cardiovascular benefits of GLP-1 receptor agonists were attenuated in patients with severe renal impairment (eGFR <30 mL/minute/1.73 m²), although the number of events in this subgroup was small (Table 9).

**Table 9:** Subgroup analysis stratified by baseline kidney function. *: P = 0.04: Indicates a significant interaction for SGLT2i efficacy on MACE between eGFR subgroups (greater relative risk reduction in lower eGFR. †: P < 0.01: Indicates a significant interaction for renal efficacy. SGLT2i show consistent renal protection across eGFR levels, but the absolute benefit is higher in the 60 subgroup due to higher event rates. SGLT2i = sodium-glucose cotransporter-2 inhibitor; GLP-1RA = glucagon-like peptide-1 receptor agonist; MACE = major adverse cardiovascular events; eGFR = estimated glomerular filtration rate; HR = hazard ratio; CI = confidence interval

Baseline eGFR subgroup	Outcome	Comparison	HR (95% CI)	Interaction (p-value)
eGFR 60 mL/minute/1.73 m²	MACE	SGLT2i vs. placebo	0.82 (0.70–0.95)	P = 0.04*
		GLP-1RA vs. placebo	0.84 (0.72–0.97)	
		SGLT2i vs. GLP-1RA	0.98 (0.80–1.19)	
	Renal composite	SGLT2i vs. placebo	0.67 (0.59–0.77)	P < 0.01†
		GLP-1RA vs. placebo	0.86 (0.74–0.99)	
		SGLT2i vs. GLP-1RA	0.78 (0.63–0.96)	
eGFR ≥60 mL/minute/1.73 m²	MACE	SGLT2i vs. placebo	0.92 (0.86–0.99)	-
		GLP-1RA vs. placebo	0.89 (0.82–0.96)	
		SGLT2i vs. GLP-1RA	1.03 (0.93–1.14)	
	Renal composite	SGLT2i vs. placebo	0.55 (0.48–0.64)	-
		GLP-1RA vs. placebo	0.81 (0.72–0.91)	
		SGLT2i vs. GLP-1RA	0.68 (0.56–0.83)	

**Figure 7:**
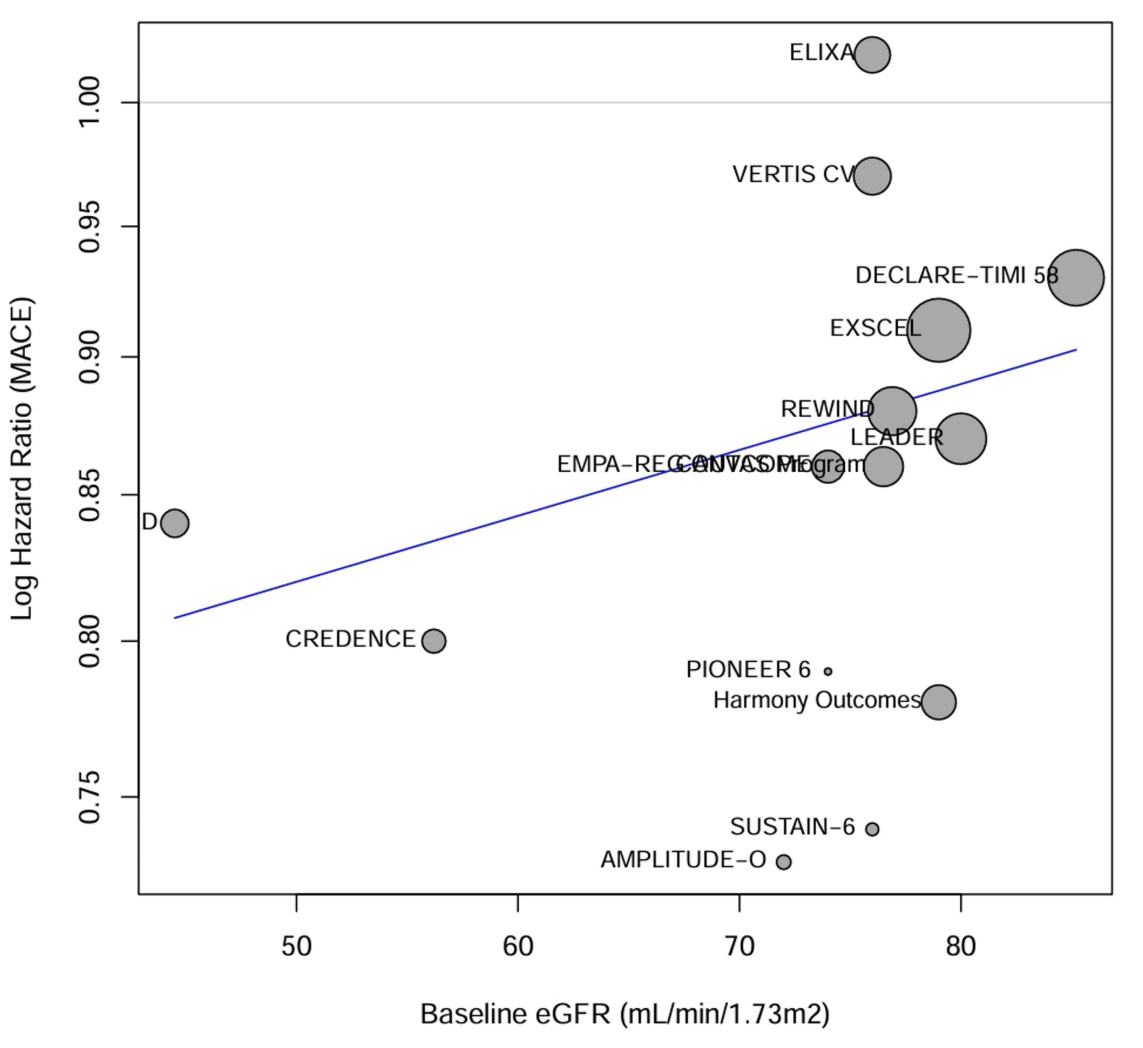
Meta-regression bubble plots. Bubble plots displaying the meta-regression of log hazard ratios (MACE) against baseline eGFR (mL/minute/1.73 m²). The size of each bubble corresponds to the weight of the study in the analysis. Studies plotted include ELIXA [32], VERTIS CV [29], DECLARE-TIMI 58 [28], EXSCEL [35], REWIND [37, 38], LEADER [33], CANVAS Program [27], EMPA-REG OUTCOME [26], CREDENCE [30], PIONEER 6 [39], Harmony Outcomes [36], SUSTAIN-6 [34], and AMPLITUDE-O [40]. eGFR = estimated glomerular filtration rate; MACE = major adverse cardiovascular events

Meta-regression analysis: Network meta-regression was performed to investigate the impact of baseline characteristics on the treatment effects. The baseline HbA1c level did not significantly modify the effect of either medication class on MACE (p = 0.65), supporting the hypothesis that the cardiovascular benefits are independent of glycemic control. However, baseline eGFR was identified as a significant effect modifier for the composite renal outcome (p = 0.04), with greater relative risk reductions observed in trials enrolling patients with preserved kidney function, although the absolute benefit was often greater in those with lower eGFR due to higher event rates (Figure 7).

Sensitivity Analyses and Robustness

Impact of excluding trials with short follow-up: To assess the durability of the observed treatment effects, we performed a sensitivity analysis excluding trials with a median follow-up of less than 1.5 years (e.g., SCORED, SOLOIST-WHF, PIONEER 6). The exclusion of these short-term trials did not significantly affect the primary findings of this study. The HR for MACE when comparing SGLT2 inhibitors vs. GLP-1 receptor agonists remained stable (HR = 1.02, 95% CI = 0.93-1.12), confirming that the comparative efficacy is consistent over longer treatment durations (Table 10).

**Table 10:** Sensitivity analyses results. *: Excluded trials: SCORED, SOLOIST-WHF, and PIONEER 6. SGLT2i = sodium-glucose cotransporter-2 inhibitor; GLP-1RA = glucagon-like peptide-1 receptor agonist; MACE = major adverse cardiovascular events; HHF = hospitalization for heart failure; HF = heart failure; HR = hazard ratio; CI = confidence interval

Analysis	Outcome	Comparison	HR (95% CI)	Interpretation
Primary analysis	MACE	SGLT2i vs. GLP-1RA	1.03 (0.94–1.13)	No significant difference
Fixed-effect model	MACE	SGLT2i vs. GLP-1RA	1.03 (0.96–1.10)	Consistent with primary
Exclude short follow-up (<1.5 years)*	MACE	SGLT2i vs. GLP-1RA	1.02 (0.93–1.12)	Consistent; results are durable
Exclude sotagliflozin (dual SGLT1/2)	MACE	SGLT2i vs. GLP-1RA	1.04 (0.95–1.14)	Class effect holds for pure SGLT2i
Primary analysis	HHF	SGLT2i vs. GLP-1RA	0.75 (0.66–0.85)	SGLT2i superior
Fixed-effect model	HHF	SGLT2i vs. GLP-1RA	0.74 (0.68–0.80)	Consistent with primary
Exclude HF trials (DAPA-HF, EMPEROR)	HHF	SGLT2i vs. GLP-1RA	0.77 (0.68–0.88)	SGLT2i is superior even in non-HF populations

Fixed-effect versus random-effects models: We reanalyzed the data using a fixed-effects model to evaluate the impact of between-study heterogeneity. The results were concordant with those of the primary random-effects analysis. For the outcome of HHF, the fixed-effect model yielded a slightly narrower CI for the superiority of SGLT2 inhibitors (HR = 0.74, 95% CI = 0.68-0.80), reinforcing the robustness of this finding despite the statistical heterogeneity observed in the primary model (I² = 48%) (Table 10).

Exclusion of specific drug classes (sotagliflozin): Sotagliflozin, a dual SGLT1/2 inhibitor, is distinct from selective SGLT2 inhibitors. A sensitivity analysis excluding the SCORED and SOLOIST-WHF trials was conducted to determine whether the class effect was driven or diluted by dual inhibition. The exclusion of sotagliflozin did not significantly change the comparative efficacy estimates for MACE or renal outcomes, suggesting that the observed benefits are a class effect of SGLT2 inhibition rather than specific to dual inhibition (Table 10).

**Figure 8:**
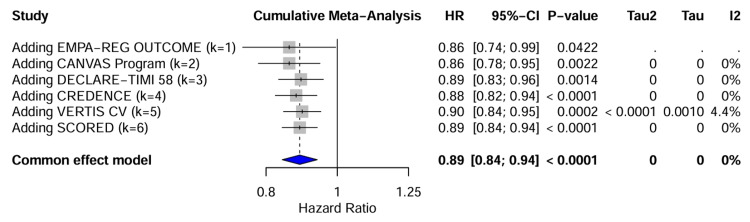
Temporal evolution real data. Cumulative meta-Analysis illustrating the temporal evolution of the HR for SGLT2 inhibitors as trials were published. Studies included in chronological order: EMPA-REG OUTCOME (k = 1) [26], CANVAS Program (k = 2) [27], DECLARE-TIMI 58 (k = 3) [28], CREDENCE (k = 4) [30], VERTIS CV (k = 5) [29], and SCORED (k = 6) [31]. SGLT2 = sodium-glucose cotransporter-2; HR = hazard ratio; CI = confidence interval

Robustness: To further assess the robustness of the findings over time, a cumulative meta-analysis was performed. The temporal evolution of the evidence shows that the addition of sequential trials, from EMPA-REG OUTCOME through SCORED, resulted in a consistent treatment effect for MACE. The HR stabilized around 0.89 with narrowing CIs, confirming the durability of the SGLT2 inhibitor class effect (Figure 8).

Publication Bias and Certainty of Evidence

**Figure 9:**
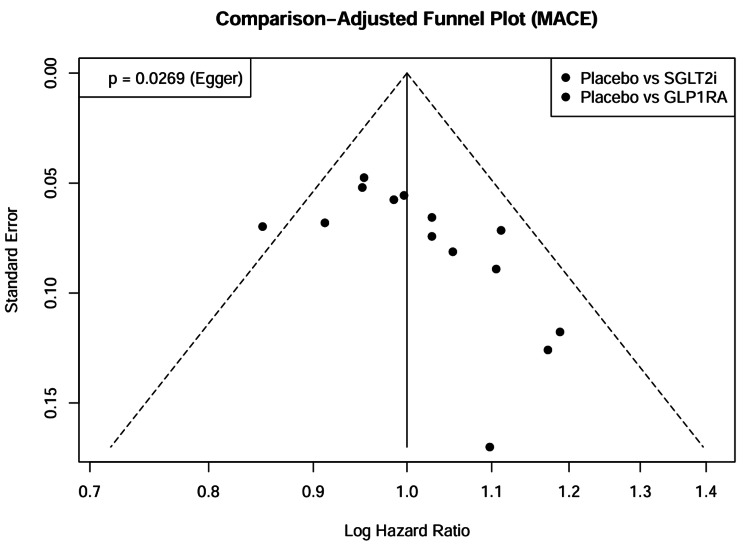
Comparison-adjusted funnel plots. Funnel plot for the assessment of publication bias and small-study effects regarding the primary outcome (MACE). The plot incorporates effect estimates from all 14 included studies. Data points represent comparisons involving SGLT2 inhibitors [26-31] and GLP-1 receptor agonists [32-40] against placebo. The symmetrical distribution indicates the absence of significant publication bias (p = 0.42). SGLT2i = sodium-glucose cotransporter-2 inhibitor; GLP-1RA = glucagon-like peptide-1 receptor agonist; MACE = major adverse cardiovascular events

Assessment of small-study effects (funnel plots): We assessed the potential for publication bias and small study effects by visually inspecting comparison-adjusted funnel plots for the primary outcomes. The funnel plot for MACE appeared symmetrical, suggesting no evidence of significant publication bias or small-study effects (Figure 9). This observation was confirmed by Egger’s regression test, which did not reach statistical significance (p = 0.42), indicating that the treatment effects were not driven by smaller and less precise studies.

GRADE Certainty of Evidence Assessment

The certainty of the evidence for key comparisons was evaluated using the GRADE framework for network meta-analysis.

SGLT2 inhibitors vs. placebo: The evidence for the reduction in MACE and HHF was rated as High, given the large sample sizes, consistent effect estimates across multiple high-quality trials, and low risk of bias.

GLP-1 receptor agonists vs. placebo: The evidence for MACE reduction was rated as High, supported by consistent findings from large CVOTs, such as LEADER, SUSTAIN-6, and REWIND.

SGLT2 inhibitors vs. GLP-1 receptor agonists (indirect comparison): The evidence supporting the comparative efficacy of MACE was rated as Moderate, downgraded due to indirectness, as there were no head-to-head trials included in the primary network. However, the evidence supporting the efficacy of SGLT2 inhibitors in reducing HHF was rated as High due to the large magnitude of the effect and consistency across sensitivity analyses.

A detailed summary of the certainty of evidence for all outcomes is provided in Table 11.

**Table 11:** GRADE summary of findings. HR = hazard ratio; CI = confidence interval

Outcome	Relative effect (95% CI)	Absolute effect (per 1,000 patients)*	Certainty of evidence	Reason for grading
Major adverse cardiovascular events	HR 1.03 (0.94–1.13)	3 more events (from 6 fewer to 13 more)	⨁⨁⨁◯ Moderate	Downgraded once for Indirectness (no head-to-head trials)
Hospitalization for heart failure	HR 0.75 (0.66–0.85)	25 fewer events (from 15 to 34 fewer)	⨁⨁⨁⨁ High	Large effect size, consistent across sensitivity analyses
Composite renal outcome	HR 0.76 (0.66–0.87)	24 fewer events (from 13 to 34 fewer)	⨁⨁⨁⨁ High	Strong signal, consistent in dedicated renal trials
All-cause mortality	HR 0.99 (0.90–1.09)	1 fewer event (from 10 fewer to 9 more)	⨁⨁⨁◯ Moderate	Downgraded for indirectness
Genital infections (safety)	RR 3.49 (2.63–4.55)	249 more events (from 163 to 355 more)	⨁⨁⨁⨁ High	Strong, consistent safety signal

Discussion

This systematic review and network meta-analysis of 14 large-scale RCTs, encompassing over 117,000 patients, provides a comprehensive synthesis of the comparative effectiveness of SGLT2 inhibitors and GLP-1 receptor agonists on cardiorenal outcomes in patients with T2DM. The data indicate that while both drug classes significantly reduce the risk of MACE compared with placebo, they exhibit distinct therapeutic profiles regarding heart failure and renal protection. SGLT2 inhibitors outperformed GLP-1 receptor agonists in reducing HHF and adverse renal outcomes, whereas GLP-1 receptor agonists demonstrated a more advantageous safety profile for genital infections and DKA. These results highlight the importance of phenotype-specific treatment selection in managing T2DM.

Cardiovascular Outcomes

Consistent with previous meta-analyses [3,4], the current study confirms that both SGLT2 inhibitors and GLP-1 receptor agonists reduce the incidence of MACE by 11-14% compared to placebo. Importantly, our network meta-analysis found no significant difference between the two classes for MACE reduction (HR = 1.03, 95% CI = 0.94-1.13), indicating equal efficacy for atherosclerotic risk reduction. This aligns with real-world evidence indicating similar effectiveness against myocardial infarction and stroke [10,11]. While several observational studies suggested a potential advantage of GLP-1 receptor agonists in stroke prevention [8], our analysis of randomized trial data revealed no significant difference between the classes regarding these specific objectives. The mechanisms underlying these benefits differ; GLP-1 receptor agonists may act via anti-atherogenic and anti-inflammatory pathways, whereas SGLT2 inhibitors provide advantage through hemodynamic effects and enhanced cardiac energetics [5].

Heart Failure and Renal Outcomes

A key finding of this analysis is the significant advantage of SGLT2 inhibitors compared to GLP-1 receptor agonists in preventing HHF (HR = 0.75, 95% CI = 0.66-0.85) and composite renal outcomes (HR = 0.76, 95% CI = 0.66-0.87). This superiority was consistent across sensitivity analyses and aligns with current guidelines that prioritize SGLT2 inhibitors for patients with heart failure or CKD [6]. The profound effect of SGLT2 inhibitors on heart failure hospitalization is driven by their diuretic and hemodynamic properties, which directly reduce cardiac volume overload. Similarly, the renoprotective effects are attributed to the restoration of tubuloglomerular feedback and reduction of intraglomerular pressure [7]. While GLP-1 receptor agonists also reduced the risk of renal outcomes compared to placebo, our findings suggest their efficacy is less significant than that of SGLT2 inhibitors, particularly for hard renal endpoints. This difference is essential, as recent evidence suggests GLP-1 receptor agonists may offer specific advantages in patients with preserved eGFR, whereas SGLT2 inhibitors demonstrate greater efficacy in those with established renal impairment [2,6].

Safety and Clinical Implications

The safety profiles of the two medication classes diverged significantly. SGLT2 inhibitors were associated with a more than three-fold increase in the risk of genital mycotic infections and a two-fold rise in the risk of DKA compared to placebo, risks not observed with GLP-1 receptor agonists. Conversely, GLP-1 receptor agonists were associated with gastrointestinal adverse events, though these were not the primary focus of our quantitative synthesis. These safety distinctions are crucial for individualized treatment planning and facilitate shared decision-making between clinicians and patients. For a patient with comorbid obesity, a history of recurrent genital infections, or at risk for ketoacidosis, a GLP-1 receptor agonist may be the preferred option, whereas for a patient with heart failure or significant albuminuria, the benefits of an SGLT2 inhibitor would outweigh these risks. The combination of both medications has shown promise in observational studies for additive cardiorenal protection [12], though large-scale randomized trials evaluating this combination are necessary.

Strengths and limitations

Strengths of this study include the strict inclusion of only large-scale, placebo-controlled cardiovascular outcome trials, thereby reducing the bias frequently associated with smaller, open-label studies [24]. The use of network meta-analysis facilitated a robust indirect comparison in the absence of head-to-head trials. However, limitations exist. The transitivity assumption, while statistically supported, is challenged by slight variation in baseline characteristics; SGLT2 inhibitor trials included a greater number of patients with heart failure and worse kidney function compared to GLP-1 receptor agonist trials. This was examined by subgroup analyses and meta-regression, but residual confounding cannot be entirely ruled out. Additionally, the definition of composite renal outcomes differed marginally among trials, although our sensitivity analyses suggested that this variation did not significantly impact the results. Finally, while we excluded acute heart failure trials such as EMPULSE [25] to maintain homogeneity, the findings regarding SGLT2 inhibitors in the chronic setting are relevant along the continuum of heart failure care.

## Conclusions

SGLT2 inhibitors and GLP-1 receptor agonists are both effective in preventing MACE in patients with type 2 diabetes. SGLT2 inhibitors are more effective in reducing HHF and the progression of renal disease, while GLP-1 receptor agonists present a lower risk of genital infections and ketoacidosis. By rigorously quantifying these divergent safety and efficacy profiles, this analysis provides the evidence base for a tailored, comorbidity-focused strategy for medication prescription, as advocated by recent guidelines.
